# Health and disease as practical concepts: exploring function in context-specific definitions

**DOI:** 10.1007/s11019-021-10058-9

**Published:** 2021-11-16

**Authors:** Rik van der Linden, Maartje Schermer

**Affiliations:** Department of Medical Ethics, Philosophy and History of Medicine, Erasmus MC Academic University Hospital, Rotterdam, The Netherlands

**Keywords:** Health, Disease, Context-specific definition, Conceptual pluralism, Pragmatism, Philosophy of medicine

## Abstract

Despite the longstanding debate on definitions of health and disease concepts, and the multitude of accounts that have been developed, no consensus has been reached. This is problematic, as the way we define health and disease has far-reaching practical consequences. In recent contributions it is proposed to view health and disease as practical- and plural concepts. Instead of searching for a general definition, it is proposed to stipulate context-specific definitions. However, it is not clear how this should be realized. In this paper, we review recent contributions to the debate, and examine the importance of context-specific definitions. In particular, we explore the usefulness of analyzing the relation between the practical function of a definition and the context it is deployed in. We demonstrate that the variety of functions that health and disease concepts need to serve makes the formulation of monistic definitions not only problematic but also undesirable. We conclude that the analysis of the practical function in relation to the context is key when formulating context-specific definitions for health and disease. At last, we discuss challenges for the pluralist stance and make recommendations for future research.

## Introduction

Despite the longstanding debate within the philosophy of medicine, consensus on definitions of health and disease concepts has not been reached. Decades of academic work have led to the development of numerous theoretical accounts, representing many different schools of thought. Depending on the account that is taken to consideration, the relation between ‘health’ and ‘disease’ can also be described in different ways: dichotomous, spectral, overlapping, or mutually exclusive. Indeed, defining health and disease appears to be very complex. At the same time, the traditional medical-philosophical debate on this issue appears to have reached deadlock—it has become stuck in polarization between naturalism and normativism. This is problematic, as the way in which we conceptualize ‘health’ and ‘disease’ has far-reaching practical consequences. Crucial decisions, such as on the inclusion in clinical trials, treatment regimens for patients, implementation of health policy measures, or eligibility for sickness benefits, all depend on the definitions used. Moreover, due to medical, technological and societal developments the landscape of health and disease is rapidly changing, challenging some of the traditional definitions. Therefore, even after decades of scholarship, the need for clear definitions has not become any less relevant.

Within the debate on health and disease concepts, we currently witness some tentative movements in what we consider a pragmatic direction. Increasingly, attention is paid to plurality, complexity and contextuality of notions of health and disease (De Vreese 2017; Haverkamp et al., 2018; Nordby 2006; Schwartz 2007, 2017; Walker & Rogers 2018). Instead of continuing with searching for monistic and general definitions, it is proposed to continue the debate by looking for stipulative and contextual definitions. This pragmatic approach implies that a theory of health and disease is not considered successful due to its correspondence to the world in a metaphysical or analytical sense but is rather viewed as successful because its correspondence to our social world, through its practical usefulness. We are sympathetic for this new pragmatic direction but also acknowledge that it comes with certain challenges. Although there are good reasons why we should look for context-specific definitions, it is not so clear yet *how* we should achieve this.

In this paper, we will describe the shift towards stipulating context-specific definitions, rather than general monist ones, and explore how we can best proceed in this direction. We suggest that taking the function of disease and health concepts and their context into account is a promising way to go. First, we briefly discuss recent contributions to the debate on health and disease concepts and present current proposals for specifying contextual definitions. Subsequently, we will explore the relation between a health or disease definition, its practical function, and the context it is deployed in—and discuss how this could be relevant in further research aimed at formulating context-specific definitions. Lastly, we will discuss challenges of this new direction and make recommendations for future research.

## Problems with monist definitions

In the traditional philosophical debate on defining health and disease, scholars have typically tried to formulate theories on monistic grounds, in which definitions have been proposed as being able to correctly identify all conditions as healthy or diseased. Well-known theories including—but certainly not limited to—Christopher Boorse’s Biostatistical Theory (1977, 2014), Lennart Nordenfelt’s Holistic Theory of Health (1995, 2007), and Jerome Wakefield’s Harmful Dysfunction account (1992), have triggered decades of academic debate, questioning which definition could correctly describe the concepts ‘health’ and ‘disease’. In recent contributions, however, scholars have provided a variety of arguments to explain why this search for monist definitions of health and disease is not likely to succeed (De Vreese 2017; Lemoine 2013; Nordby 2006; Schwartz 2017), which has radical implications for the continuation of the debate.

In Nordby (2006), it is argued that conceptual analysis cannot yield a pure conceptual truth about our common health concepts (i.e. disease, illness, sickness). The assumption of conceptual analysis is that there *is* a definition of the target concept to be found, and that this is not merely a stipulative definition but one that constitutes a general normative standard. Nordby invokes the analytic-synthetic argument to show that this assumption, which is based on a form of semantic realism, is incorrect. He argues that concept definitions, including definitions of disease, are never analytic, i.e. true purely in virtue of meaning. Therefore, conceptual analysis cannot succeed in formulating a general definition that is a correct articulation of some underlying ‘true’ meaning of the concept of disease.^1^ The many different meanings of health and disease concepts, as used by different speakers and in different contexts, cannot be captured by a single definition.

In line with Nordby (2006), Schwartz (2007, 2017) claims that it is not possible to discover a purely analytical definition of the disease concept, which consequently renders the use of conceptual analysis to be ‘‘deeply problematic’’. Besides drawing on several important works in the philosophy of language, Schwartz also turns to empirical research to substantiate his claim. Firstly, he argues that the classical view of concepts (i.e., where concepts are represented by a list of necessary and sufficient conditions) is undermined by psychological research, in which is demonstrated that objects are usually classified on basis of characteristics that are not strictly necessary. Secondly, he explains that research also shows that the way people classify objects is not a dichotomous process (‘all-or-nothing’’), as is the case for the classical view of concepts, but is rather a matter of degree. Finally, Schwartz observes that people actually use the term ‘disease’ in different ways. He explains that there is not only an apparent disparity in the way medical doctors and laypeople use the term, but also a single person may use the term in different ways, at different times.

Lemoine (2013) has not explicitly argued against conceptual monism. At least, not by discussing it as a theoretical impossibility. However, instead, he focuses on the practical impossibility and argues that conceptual analysis is not up to the task of delivering such a definition. By analyzing Boorse (1977), Nordenfelt (1995), and Wakefield (1992) he concludes that there is a serious flaw in the use of this method. That is, scholars are free to choose the ‘‘set of descriptive predicates in order to draw the right lines between cases of ‘health’ and cases of ‘non-health’ on the one hand, and cases of ‘disease’ and cases of ‘non-disease’ on the other.’’ (Lemoine 2013, p. 24). This means that their definitions may (in theory) successfully describe the extensions they refer to, but that these extensions clearly vary. The only way to end up with one successful definition of disease is then to show that the competing definitions are based on extensions that are incorrect (i.e. demonstrating that they include conditions that are not diseases or exclude conditions that are diseases). However, this appears to be a normative decision, which cannot be decided upon by using conceptual analysis. In conclusion, Lemoine argues that it appears that scholars often talk past each other while presenting counterarguments.

In De Vreese (2017), finally, some other interesting arguments are provided to explain why the search for a monistic definition is unlikely to be successful. De Vreese also criticizes the use of conceptual analysis, but for a different reason than Lemoine and Schwartz. Instead of presenting concerns regarding epistemological issues with conceptual analysis, De Vreese’s arguments primarily addreses the plural nature and structure of the disease concept. De Vreese, drawing on Haslam (2002), claims that the concept ‘disease’ does not refer to one specific kind but to several: *natural kinds, discrete kinds, fuzzy kinds, spectral kinds,* and *non-kinds*. The complex non-classical structure of ‘disease’ would be incompatible with a monist definition that assumes a classical structure. Furthermore, De Vreese argues that ‘disease’ should be viewed as a practical concept that has varying meanings, is intrinsically value-laden, and which use is inevitably influenced by developments in medicine (2017, p. 429). This makes it very difficult, if not impossible, to arrive at monistic definitions. Hence, De Vreese proposes to continue the debate by taking a pragmatic approach.

In summary, recent contributions point at various problems that come with establishing one general, overarching definition. The dynamic nature and contextuality of concepts, and that of health and disease concepts in particular, makes it difficult to capture all different meanings in one single definition. In addition, epistemological issues concerning the use of conceptual analysis further challenge the search for monistic definitions. Frequently, in the traditional debate, a definition has been criticized for being unable to capture all conditions that are considered healthy or diseased. However, if it is in fact impossible to formulate a successful monist definition, such disputes are fruitless. Rather, it appears that scholars often talk past each other, creating a discussion that sometimes could be considered as a ‘meta-linguistic negotiation’^2^ (Plunkett, 2015). When all these arguments are taken into consideration, it appears that the search for monistic definitions is indeed deeply problematic, if not, untenable.

## Conceptual pluralism

In trying to overcome the problems of monistic definitions, scholars have proposed to consider health and disease as plural concepts instead. To better understand the diversity in meaning of health and disease concepts, Nordby (2006, 2019), for instance, proposes to consider Wittgenstein’s philosophy as a theoretical foundation. Referring to Ludwig Wittgenstein (1953), he remarks that health and disease concepts are controversial because they are used in a variety of different ‘language games’ with various implicit and explicit rules. This can be problematic as speakers of one language game are often not willing to adjust or conform to the rules of another language game (Nordby 2019).

Nordby (2006) first suggests that despite this plurality the search for definitions could still be continued, but in alternative ways. One might arrive at a general definition by demonstrating why one particular ‘language game’ is more correct than another. However, conceptual issues (about meaning and understanding) are not subject to questions of objectivity in the way epistemological issues (about truth and knowledge) are. It is commonly accepted that different speakers can understand and use a word in different ways. Hence, Nordby argues we should remain skeptical to the idea of finding general definitions by this alternative method. Instead, he proposes to look for stipulative and/or contextual definitions that fit the assumptions about correct usage of particular areas or domains, that is: within particular language games.

Whereas Nordby (2006, 2019) proposes to formulate stipulative and contextual definitions by examining actual usage within certain areas, Schwartz (2007, 2017) proposes to take a different path. He remarks that conceptual analysis can only be used to analyze the current use and meaning of a concept, but not how a concept *ought* to be used.^3^ Drawing on Carnap rather than Wittgenstein, he proposes for using what he calls ‘philosophical explication’, which means that: ‘‘the clarification of the concept of “disease” is not discovered, but instead is set, through the careful definition of a new term that can play the role of the old one.’’ (Schwartz, 2017, p. 496). Instead of examining how concepts are currently used within a group of speakers, Schwartz claims it is more useful to take a forward-looking approach. For Schwartz, stipulating a definition is not achieved by looking at the current use of the concept but rather is a process of explicating what kind of new definition is needed in light of a particular problem that needs solving. He notes that any new definition will impose some changes and may come with counter-intuitive consequences. Moreover, he explains that there may be a need for different definitions for different contexts.

Echoing Nordby (2006), Walker & Rogers (2018) argue that health and disease concepts should be viewed as being connected through Wittgensteinian family resemblances. Interestingly, however, Walker & Rogers argue that although a ‘classical’ monistic definition of health and disease cannot be formulated, it may still be possible to generate a general *cluster-concept*. They explain that a cluster definition allows for a plurality of ways of meeting the definition—but that the cluster-concept does not itself imply plural definitions. We agree and believe that a cluster-concept could in fact be described as a form of *fuzzy monism.* However, Walker & Rogers note that such a general cluster-concept is too vague to be practically useful. Therefore, they propose that we need to distinguish more specific definitions as ‘précisifications’ within the broader cluster-concept.

Whereas it is very clear why we need ‘précising definitions’, it is not so clear why we would still want to have the very broad and vague general definition that a cluster concept would provide. Defining a cluster-concept seems to be primarily aimed at meeting the needs of plurality and at the same time satisfying (to some degree) scholars that defend conceptual monism. Reasoning from a pragmatist perspective, however, we may eventually be better off to stop quibbling about the exact conceptual structure of health and disease concepts, as long as it does not appear to make any difference in practice. Instead, we propose to shift the focus towards the question of how context-specific definitions could be successfully formulated.

## Context-specific definitions

As we have shown in the previous sections, the new direction of the debate on health and disease concepts is clear. At least, in theory. However, what is not so clear from the contributions as discussed so far, is *how* we should proceed. Although the arguments and proposals we discussed justify the search for a plurality of stipulative and contextual definitions of health and disease, it does not tell us anything about the types of contexts we should aim for—nor do they provide us with a clear method or strategy for stipulation and explication. Fortunately, however, some scholars have already made valuable proposals.

In a recent contribution to the debate, Powell & Scarffe (2019) argue that definitions of the concept of disease should be tailored to the role that the concept plays in the institutional settings in which it is deployed. They explain that: ‘‘concepts are specified in relation to institutions and are shaped by particular pragmatic, epistemic or ethical goals’’ (2019, p. 9). Moreover, they argue that these goals can differ between institutions. What exactly these goals are is not directly clear in their paper, however. Nevertheless, they explicitly argue that naturalistic theories of disease do not succeed because they do not fit the role the disease concept plays in our healthcare institutions. Instead, they propose a hybrid model for this context. Powell & Scarffe note that this hybrid model may not be useful for other sciences that make use of the disease concept and remark that:

‘‘Theoretical unification is a worthwhile scientific pursuit, and since human medicine may reasonably be viewed as a subset of biological science, one might argue that we should aim for concepts that unify the medical and biological domains. Yet, a concept of disease that is useful in, say, veterinary medicine or forestry science may be poorly suited to the thickly normative aims of human medicine. Furthermore, the moral institutional dimensions of the disease concept are not limited to matters of healthcare allocation.’’ (2019, p. 9)

Interestingly, although Powell & Scarffe defend the pluralist stance, they do not advocate a pluralistic approach to disease *within* the context of healthcare. Instead, they argue in favor of conceptual unity in this context. This means a plurality of meanings is accepted, but only between disciplines. In the philosophical literature, this is sometimes also referred to as *between-discipline* pluralism—which is the opposite of *within-discipline* pluralism (Garson, 2018).

We agree with Powell & Scarffe that the concepts should be tailored to the ‘role’ the concepts serve in institutional settings, and we can also imagine that there might be a need for an institution-broad definition of disease. Such a broader definition could be used as a conceptual tool for communicating health policy within or between health care organizations, for example, by steering medical practice from curing diseases towards prevention and lifestyle medicine. However, what remains unclear in the proposal by Powell & Scarffe is what is meant with a ‘role’ and why it is necessary or useful to view ‘healthcare’ as being one institution. Healthcare is a complex enterprise that is interdependent on clinical practice, medical sciences, (pharmaceutical) industry, health insurers, politics and economic institutions, et cetera. If health and disease concepts should indeed be tailored to the role they play in different settings, it seems insufficient to take healthcare as being one institution that can function with one single definition.

In another recent contribution, Haverkamp and colleagues (2018) argue that health concepts are practice-oriented. They state that the search for a health concept can guide particular health practices in reflecting on their goals and in formulating their priorities. The suggestion to look at practices instead of institutions seems to be a good idea, as it could distinguish between the various aspects within the broader healthcare institution. Haverkamp et al. argue that the values that are important within a certain practice and that are action-guiding within that practice should be coherent with the health concept of such practice. For example, in care for chronically ill patients, the subjective experience and well-being of patients is deemed important. Therefore, a suitable health concept to guide this practice, should include the subjective point of view of the patient and relate health to well-being. Definitions by Nordenfelt (1993a) or Huber et al. (2011) are considered possible candidates. Biomedical research, on the other hand, ‘‘given its scientific character’’ (p. 396), may be served better by a scientific definition of health, such as formulated by Boorse, they claim. Another health practice they discuss is that of public health policy, in particular in relation to health inequities. To measure inequality of health levels it seems preferable that health is understood in an objectivist sense. However, to account for the diversity in societal norms and values of a particular society, a circumstantialist health concept (e.g. as proposed by Venkatempuram (2011)) may be best suited to promote public health.

Although the proposal by Haverkamp et al. is interesting because it provides a more detailed picture of the possible needs of different healthcare practices, it is not always clear *why* a particular context (i.e. practice) would need a specific definition. For example, it seems a bit circular to argue that biomedical research may benefit from a ‘scientific definition’ because of its ‘scientific character’. Furthermore, the practices mentioned by Haverkamp et al. are still quite broad categories, consisting of many different subfields. It is imaginable that a nanobiologist could need a different definition of health/disease than a health scientist, while both professions may be categorized as biomedical research. Thus, it appears that this way of making top-down recommendations may paradoxically lead to a more static philosophical understanding of what is considered health and disease within one broad field of practice. Therefore, more (sub)specifications may be required. On the other hand, different practices within the healthcare institution have to be able to communicate with each other and work together, which might be a challenge with accepting all these practice-oriented definitions. Haverkamp et al. recognize this problem and therefore also question if an integrated approach would not be preferable. However, an integrated approach, they argue, will fail because of the problems with monist definitions that we discussed in the previous sections.

If we follow up on these suggestions to formulate different definitions for different contexts, it is important to specify clearly *why* a particular context needs a specific definition. Walker & Rogers (2018), drawing on Kingsbury and McKeown-Green (2009), link this to the idea that definitions should be *motivationally adequate*. This means that a definition should correspond with the reasons we have for wanting to put conditions together as a class rather than a collection of separate items. They explain:

‘‘A definition is motivationally adequate when it is “justified” in relation to there being some practice or theory that makes sense of why we want to group the items in the category together.’’ (Walker and Rogers, 2018, p. 415).

Walker & Rogers further explain that we have both theoretical and practical reasons for particular groupings. For example, we might want to facilitate studying certain types of disease such as genetic ones, or we might want to group conditions together for purposes of arranging efficient healthcare delivery systems. This implies that definitions of health and disease may differ depending on the motivation for grouping conditions together as healthy or diseased. Importantly, motivational adequacy asks for reasons for using specific definitions in specific contexts. In Powell & Scarffe (2019) this motivation is not very clearly articulated, and it is still a bit vague in Haverkamp et al. (2018). We propose—as Walker and Rogers implicitly seem to do as well—to look at these reasons in terms of the role or function that we expect a definition to fulfil in a particular context. Therefore, it seems useful to explore the specific function(s) that health and disease definitions are expected to serve in particular contexts.

## Exploring function in context-specific definitions

In the academic debate, various reasons have been given for the need of health and disease definitions. In a broad sense, definitions of health and disease can help to delineate the purposes or aims of specific practices and can be ‘action guiding’ in that they emphasize certain aspects and values that are deemed important. More concretely, health and disease definitions guide clinical practice—they function to distinguish those in need of medical attention from those who do not. In the social domain, definitions of health and disease play an important social and economic role. A definition of disease can also be necessary to assess one’s right to receive economic benefits, exemption of social duties, and moral accountability (Nordenfelt, 1993b). Such issues are perhaps most apparent in debates about ‘‘grey cases’’—conditions whose status as diseases is controversial or intuitively unclear. Worrall & Worrall (2001) explain that the need to classify a condition as a disease often starts with practical issues, such as trying to arrange reimbursement of treatment costs. They argue that the judgements given concerning such grey cases may be often disguised as scientific matters but are in fact evaluative, political and normative matters.

As the way we define health and disease has important practical consequences, it seems useful and reasonable to take this into account when assessing what kind of definition is needed in a particular context. Therefore, in order to formulate a context-specific definition, we argue that it is important to analyze the *function* that a definition ought to serve in a particular context, as well as to look at the practical *consequences* of the definition. Walker & Rogers (2018) rightfully remark that:

‘‘A précising definition of disease, when applied to states that are borderline cases of disease, could thus sometimes appropriately refer to whether or not classification of a particular condition as a disease would have beneficial practical effects’’ (415).

In line with the idea of motivational adequacy, they argue that considering these practical aspects for the stipulation of a specified definition is justified, and actually not unusual in our assessment of definitions in general:

“Wherever there is reason to seek a definition, there is reason to require that that definition meets purposes for which it is sought.’’ (415).^4^

Considering the various functions that health and disease concepts could serve, it is reasonable to argue that one particular definition may serve a specific function better than another. To further explore this idea, we will examine a few concrete examples of the relation between a specific definition, its function and the context. It is useful to start such exploration by looking at situations in which currently used or proposed definitions are viewed as insufficient. It is in such *problematic situations*^5^ where we may gain important insights regarding the practical functions of health and disease definitions.

### Specifying disease

First, we discuss a proposal by Thomas Schramme (2007), who has made a concrete suggestion to use a specific definition of disease to serve a specific function within a particular context. Schramme argues that a clear definition of disease is especially needed—in combination with a specific theory on distributive justice—to serve as a gatekeeper for medicalization and to justify claims on health resources. In doing so, he explicitly makes a link between the function of the definition and the context. In light of the scarcity of healthcare resources, Schramme argues that a naturalist definition of health, in particular Boorse’s Biostatistical Theory (BST), should be used to narrow down the scope of what should be considered as medical conditions, and hence what should count as a legitimate claim to healthcare resources.^6^

According to Schramme (2007), a naturalist definition is necessary because a normative definition—referring to Nordenfelt’s welfare theory of health (1995; 2007) in particular—would lead to a ‘‘likely medicalization of all kinds of problems in life’’ (15), which would in turn lead to a high appeal for medical resources and the ever-increasing health care costs. Schramme has emphasized elsewhere (2019) that to make normative decisions, such as which conditions deserve publicly-funded treatment, we need a firm and objective foundation, which a naturalistic concept of disease can offer (2019, p. 13). The BST is thus not defended by Schramme as a general definition but is proposed to serve the specific purpose of limiting medicalization and a growing appeal to healthcare resources. Thus, ironically, the suggestion to use a naturalist account appears to be a normative decision itself. Schramme himself is aware of this and rightly remarks that:

‘‘The justification of specific claims on resources in health care is influenced partially by the kind of theory of disease endorsed, but it is also dependent upon which particular purpose is served by a theory of disease. It seems to me that not all possible purposes of such theories are compatible with the specific task of backing entitlements to resources. A pathologist, for example, who is interested in the functions and dysfunctions of the human organism, a doctor who writes a report on a person applying for early retirement, or a judge who needs to find a verdict on a case of a patient who sues for funding of Viagra—they are all engaged with the concept of disease in direct or indirect ways. But their different purposes seem to ask for different conceptualisations of disease.’’ (2007, p. 123)

Indeed, although one definition may be successfully used for a specific purpose, it may well be the case that other purposes may need different definitions. Whereas the BST might be used successfully for the purposes described by Schramme, the definition is considered to be insufficient and even counterproductive in some clinical contexts. For example, it has been argued that using the BST may lead to over-diagnosis and overtreatment (Rogers & Walker, 2018; Walker & Rogers 2017). Although they might agree with Schramme that a dysfunction-requirement could help to prevent expansion of the disease concept to conditions where there is no identifiable dysfunction, they stress that the BST can be problematic in other ways. When used for clarifying boundaries of diseases in clinical practice the BST may lead to overdiagnosis, because it is insufficiently clear on which level one can speak of biological dysfunction, which makes it problematic to set the threshold for pathology.

The BST appears unable to define the boundaries of a disease on a micro-level: biological abnormalities can be detected that are clinically insignificant. Doust, Walker and Rogers (2017a) therefore argue that the BST is vulnerable to what has been referred to as the *line-drawing* problem (Rogers & Walker, 2017). By providing examples regarding setting the diagnostic threshold for cancer and for infectious diseases, Walker & Rogers (2017) demonstrate that a different definition of disease is needed for the purposes of clinical medicine. They suggest a précising definition, aimed specifically at prevention of overdiagnosis. The function of this definition is to distinguish cases where it would be beneficial to identify (and treat) a condition from those where diagnosis is more likely to harm than benefit (Rogers & Walker, 2018). Although they do not specify this themselves, it appears that such a précising definition would be particularly useful in the context of screening, or in the assessment of so-called ‘incidental findings’ in clinical practice and biomedical research.

In general, where the line between the normal and the pathological should be drawn may differ between contexts. As is discussed in Doust et al. (2017b) and in Schermer & Richard (2019), for example, the line between the normal and the pathological may be drawn differently for research purposes than for clinical purposes. In some instances, it could be useful to classify a condition as pathological in a research context, while it should not be classified as a disease in clinical practice. This implies that it is not only the function of a definition that is important when stipulating a definition, but also the context it is deployed in.

To summarize, whereas the BST might be useful for certain practical purposes in the context of public policy (e.g. to serve as a gatekeeper for medicalization), it appears to be ineffective and even counterproductive for functioning in other contexts. While the BST could possibly be used to identify which conditions should be considered as diseases on a macro level (what counts as a disease), it cannot be used for line-drawing decisions on a micro level (when a specific abnormality should be considered pathological). Thus, line-drawing between health and disease, or normal and pathological, may vary between contexts and should correspond with reasons we have for drawing this line.

### Specifying health

We can do a similar exercise of functional and contextual specification for the health concept. A good example that demonstrates the relationship between function and context can be found in the heated debate on the WHO definition from 1948 that defines health as ‘‘a state of complete physical, mental, and social well-being’’ (2006). The WHO definition of health was mainly criticized for not being able to be used for scientific measurements, and for being far too broad and contributing to medicalization. Moreover, concerning the high standard of ‘complete’ well-being, the WHO definition is often viewed as too ambitious, if not idealistic and unreachable, especially as chronic diseases have become highly prevalent in our aging population. In this regard, Smith (2008) has argued that the requirement for complete health “would leave most of us unhealthy most of the time”.

While acknowledging the criticism raised against the WHO definition, Bickenbach (2015) argues that the definition was successfully used as an advocacy tool to promote international public health. Furthermore, he demonstrated that the WHO itself uses a different, more descriptive, definition of health for measurement purposes implying that they recognize the difference in function of different health concepts. Moreover, if the WHO definition is placed against the historical background, one may wonder if the definition was ever proposed to be used for matters such as doing scientific measurements and guiding clinical medicine. The WHO was initially primarily established for the purpose of promoting global (but eventually also regional, national and local) public health policy (Borowy 2014). In this sense, a broad definition that includes not only biological but also mental and social aspects of health, seems reasonable and useful.^7^

Although the WHO definition of health appears to be successfully used for public health promotion, it may indeed be less useful for other types of functions—for example, to be used for research purposes or to guide clinical medicine. From that perspective, problems of operationalizability and medicalization concerning the WHO definition were considered core reasons for Huber and colleagues (2011) to develop a new definition of health formulated as ‘‘the ability to adapt and self-manage in the face of social, physical, and emotional challenges’’. These authors point explicitly at different functions that health concepts should serve:

‘‘The general concept of health is useful for management and policies, and it can also support doctors in their daily communication with patients because it focuses on empowerment of the patient (for example, by changing a lifestyle), which the doctor can explain instead of just removing symptoms by a drug. However, operational definitions are needed for measurement purposes, research, and evaluating interventions.’’ (2).

So, interestingly, Huber et al. differentiate between the use of a general concept of health and the use of various operational definitions. While a ‘general’ definition^8^ could function as a conceptual tool for daily communication between doctor and patient, operational definitions are needed to serve measurement purposes in scientific research. If these measurement purposes can be actually achieved by implementing this new definition is still up for debate, however (Prinsen & Terwee 2019). Furthermore, in later work, Huber et al. also stress other functions of the new concept of ‘positive health’ as she calls it. In the context of clinical medicine, it is said to empower patients and directs physicians’ attention to the resilience and adaptive capacities of their patients. In the field of health policy-making, it should bridge the gap between different institutional domains, like social welfare and medicine (Huber et al. 2016).

This example of defining the health concept, as well as the example of defining the disease concept, elucidates that it is not sufficient (or perhaps even not possible), to consider the functions or purposes of a definition in isolation. A function is deployed in a specific context and the context is also specified by boundaries of the use of the concept. For example, measuring health could be viewed as a function that is typically deployed in a research context, or the health-policy context, but not so much in the context of public health promotion, or clinical medicine. The function is bounded by its context. On the other hand, to specify the context is to look where a specific function is needed and to explore why this is the case. This implies that we could look for specific practice-oriented definitions, but also for institution-broad definitions, and everything in between. It is the relation between a function and the context it is deployed in that makes a context-specific definition meaningful, not the context nor the function by itself.

## Challenges and the way forward

As we have demonstrated in this paper, health and disease concepts ought to serve various practical functions, in various contexts. It is therefore not only very unlikely that we will arrive at monist definitions, but also—and perhaps primarily—not desirable. Although, as we have argued, accepting a plurality of health and disease concepts is not problematic in itself, it does pose certain challenges. One should, first of all, be careful when extrapolating a context-specific definition beyond its proposed application. One may rightfully criticize a particular definition for not being suitable to a specific function and/or context, but it would be incorrect to conclude that this means that the definition is invalid or unsuccessful per se. This also implies that well-known definitions such as those by Boorse or Nordenfelt might still be relevant, in as far as they can be demonstrated to serve a particular function in a specific context. They should, however, no longer be thought of as monistic definitions, providing the one and only right conceptualization of disease. That being said, demonstrating that a certain definition may or may not be useful for a particular function or context constitutes a valuable contribution to the debate, as it clarifies the limitations and boundaries of a concept—as we did, in our brief analysis of several specific health and disease definitions.

Secondly, when different definitions are proposed and used for different purposes and in different contexts, communication across domains may become more challenging. Working with a plurality of health and disease concepts may raise confusion where contexts meet, or when multiple functions are at stake. Moreover, contexts may overlap to some extent. Not only because different fields or practices may have similar values, aims, and priorities, but also because they are sometimes connected or interdependent. In this case, more general or overarching definitions could be needed. Here, a Wittgensteinian view of health and disease concepts may prove to be useful as a conceptual tool to understand how different definitions (‘language games’) may exist alongside each other but also occasionally overlap.

Finally, while we have argued that we should take the function and context of use of a definition into account when specifying health and disease definitions, our analysis does not yet provide clear-cut solutions for the challenge of stipulating such context-specific definitions.

However, to successfully formulate context-specific definitions, we believe that it is important to take a pragmatic ‘bottom-up’ approach by departing from actual practice, since it may be impossible to say beforehand where the focus and locus of defining health and disease must lie. This should arise from practical necessity, not from philosophical loftiness. In this paper, we have only focused on some specific theoretical proposals, without exploring the actual use in practice. An empirical analysis may contribute to a more detailed picture of the specific functions that health and disease concepts actually serve in practice and which definitions are deployed in specific contexts. Important insights could also be gained by examining how disease definitions have changed through history and for what reasons they have changed, or by exploring what kind of definitions are emerging around new medical-technological developments. With this, we may deepen our understanding of what is useful or desirable, and what can be considered to count as adequate or successful.^9^ From a pragmatist approach, such matters only become clear by exploring the relationship between theory and practice. This approach will most likely not completely resolve all debates or lead to complete consensus, but it will help to focus the discussion on what really matters.

## Conclusion

Recent contributions to the philosophy of medicine have provided interesting ideas for proceeding the debate on health and disease concepts. By accepting conceptual pluralism, more specific health and disease concepts can be formulated by stipulation/explication—creating a palette of diverse definitions. We are sympathetic for this new pragmatic direction. However, although the theoretical necessity of having plural definitions has been made clear, scholars have not elaborated so much on the practical utility of pluralism and on how we should realize this. In this paper, we have demonstrated that health and disease concepts fulfill various practical functions, depending on the context they are deployed in. This means there is a practical need for having a plurality of health and disease definitions, and it also implies that, besides the unlikeliness of ‘discovering’ a monist definition of health and disease, this would also seem to be undesirable.

Moreover, we have argued that some definitions may serve a particular function better than others. For example, health and disease concepts that are meant to function as gatekeeper for medicalization may not be suitable to guide clinical practice or to be used for measurements in medical research. Hence, we have argued that we should analyze a definition in relation to its practical function and the particular context it is deployed in. To continue the pragmatic direction of the debate, when developing context-specific definitions, we recommend future research to depart from actual (problems in) practice. Therefore, in addition to philosophical analysis, also empirical and historical methods could be used to further explore what kind of definitions are considered important or even necessary. The adequacy and success of such definitions should ultimately be assessed through their usefulness in practice.

## Data Availability

Not applicable.

## Footnotes

1 Nordby’s argument is aimed at the semantic realism that appears to inform the quest for definitions of health and disease. While the semantic realist thinks that these concepts have real determinate extensions that a correct definition should capture, Nordby aims to argue that this assumption is false.

2 Plunkett defines a metalinguistic negotiation as a dispute that concerns a normative issue about what a concept should mean or how it should be used, but which is often perceived as a descriptive issue at first glance—resulting in a conversation in which speakers talk past each other.

3 This argument can be traced back to Schwartz’s 2004 paper on the ‘biological function’ debate, where he argued in similar spirit that it is not possible to arrive at an analytically true meaning of biological function—and hence, argued we should stop using conceptual analysis for this matter.

4 Sometimes the distinction between practical consequence and function is difficult to make. It seems unjustified to formulate a disease definition in a particular way *just* because it has some beneficial practical effect. However, when there are different legitimate definitions to choose from, looking at the consequences of choosing either one, seems justified (if not required).

5 The notion of ‘problematic situation’is derived from John Dewey’s Theory of Inquiry (1938, but see: Wright 2007), and is also employed by other pragmatist philosophers such as Philip Kitcher (2017).

6 Of course, it is open for discussion whether Schramme’s proposal to use the BST is the best option as it comes to function as gatekeeper for medicalization. Also, one could question the ‘objectivity’ of the BST (e.g., see Kingma 2007). However, these discussions would go beyond the scope of this paper.

7 Borowy notes, however, that in times of the Cold War, the WHO definition faded into the background, as the ‘social’ definition was associated with Communism (while the WHO was primarily funded by the USA). In those years, the broad WHO definition temporarily gave way to for a strong anti-disease movement, focusing primarily on the eradication of infectious diseases—as these were commonly viewed as a sign of backwardness.

8 ‘General’ is used by Huber et al. as having a broader function, but is not general in the sense that it overarching, universally applicable, or monistic.

9 We thank an anonymous reviewer for pointing us to MacIntyre’s concept of ‘practice’, which distinguishes between *internal goods* and *external goods*. It seems worthwhile to explore if the assessment of *motivational adequacy* may benefit from this framework. For example, we could state that a morally good motivation must always improve aspects that we consider to be internal goods for medicine.
